# Healthcare workers hospitalized due to COVID-19 have no higher risk of death than general population. Data from the Spanish SEMI-COVID-19 Registry

**DOI:** 10.1371/journal.pone.0247422

**Published:** 2021-02-19

**Authors:** Jesús Díez-Manglano, Marta Nataya Solís-Marquínez, Andrea Álvarez García, Nicolás Alcalá-Rivera, Irene Maderuelo Riesco, Martín Gericó Aseguinolaza, José Luis Beato Pérez, Manuel Méndez Bailón, Ane-Elbire Labirua-Iturburu Ruiz, Miriam García Gómez, Carmen Martínez Cilleros, Paula María Pesqueira Fontan, Lucy Abella Vázquez, Julio César Blázquez Encinar, Ramon Boixeda, Ricardo Gil Sánchez, Andrés de la Peña Fernández, José Loureiro Amigo, Joaquín Escobar Sevilla, Marcos Guzmán Garcia, María Dolores Martín Escalante, Jeffrey Oskar Magallanes Gamboa, Ángel Luis Martínez González, Carlos Lumbreras Bermejo, Juan Miguel Antón Santos, for the SEMI-COVID-19 Network

**Affiliations:** 1 Internal Medicine Department, Royo Villanova Hospital, Zaragoza, Spain; 2 Internal Medicine Department, San Agustin University Hospital, Avilés, Asturias, Spain; 3 Internal Medicine Department, Albacete University Hospital, Albacete, Spain; 4 Internal Medicine Department, San Carlos Clinical Hospital, Madrid, Spain; 5 Internal Medicine Department, Santa Marina Hospital, Bilbao, Spain; 6 Internal Medicine Department, Urduliz Alfredo Espinosa Hospital, Urdúliz, Vizcaya, Spain; 7 Internal Medicine Department, HLA Moncloa Hospital, Madrid, Spain; 8 Internal Medicine Department, Santiago Clinical Hospital, Santiago de Compostela, A Coruña, Spain; 9 Internal Medicine Department, Nuestra Señora Candelaria University Hospital, Santa Cruz de Tenerife, Spain; 10 Internal Medicine Department, Torrevieja University Hospital, Torrevieja, Alicante, Spain; 11 Internal Medicine Department, Mataró Hospital, Mataró, Barcelona, Spain; 12 Internal Medicine Department, La Fe University Hospital, Valencia, Spain; 13 Internal Medicine Department, Son Llàtzer University Hospital, Palma de Mallorca, Spain; 14 Internal Medicine Department, Moisès Broggi Hospital, Sant Joan Despí, Barcelona, Spain; 15 Internal Medicine Department, Virgen de las Nieves University Hospital, Granada, Spain; 16 Internal Medicine Department, San Juan de la Cruz Hospital, Úbeda, Jaén, Spain; 17 Internal Medicine Department, Costa del Sol Hospital, Marbella, Málaga, Spain; 18 Internal Medicine Department, Nuestra Señora del Prado Hospital, Talavera de la Reina, Toledo, Spain; 19 Internal Medicine Department, León University Hospital, León, Spain; 20 Internal Medicine Department, 12 de Octubre University Hospital, Madrid, Spain; 21 Internal Medicine Department, Infanta Cristina University Hospital, Parla, Madrid, Spain; BronxCare Health System, Affiliated with Icahn School of Medicine at Mount Sinai, NY, USA, UNITED STATES

## Abstract

**Aim:**

To determine whether healthcare workers (HCW) hospitalized in Spain due to COVID-19 have a worse prognosis than non-healthcare workers (NHCW).

**Methods:**

Observational cohort study based on the SEMI-COVID-19 Registry, a nationwide registry that collects sociodemographic, clinical, laboratory, and treatment data on patients hospitalised with COVID-19 in Spain. Patients aged 20–65 years were selected. A multivariate logistic regression model was performed to identify factors associated with mortality.

**Results:**

As of 22 May 2020, 4393 patients were included, of whom 419 (9.5%) were HCW. Median (interquartile range) age of HCW was 52 (15) years and 62.4% were women. Prevalence of comorbidities and severe radiological findings upon admission were less frequent in HCW. There were no difference in need of respiratory support and admission to intensive care unit, but occurrence of sepsis and in-hospital mortality was lower in HCW (1.7% vs. 3.9%; p = 0.024 and 0.7% vs. 4.8%; p<0.001 respectively). Age, male sex and comorbidity, were independently associated with higher in-hospital mortality and healthcare working with lower mortality (OR 0.211, 95%CI 0.067–0.667, p = 0.008). 30-days survival was higher in HCW (0.968 vs. 0.851 p<0.001).

**Conclusions:**

Hospitalized COVID-19 HCW had fewer comorbidities and a better prognosis than NHCW. Our results suggest that professional exposure to COVID-19 in HCW does not carry more clinical severity nor mortality.

## Introduction

As of 30 October 2020, coronavirus disease 2019 (COVID-19), caused by severe acute respiratory syndrome coronavirus 2 (SARS-CoV-2), has affected 44,592,789 people worldwide [1]. Spain has been one of the countries with the highest number of confirmed cases and deaths.

Healthcare workers (HCW) are at high risk of infection with SARS-CoV-2 because of their exposure to infected patients. Several seroepidemiological studies have shown discordant results. In New York and Sweden the seroprevalence of SARS-CoV-2 among hospital HCW was high compared with the community [2, 3]. However, in Germany the seroprevalence in HCW was very low, 0.33% [4]. A recent systematic review and meta-analysis, that included 11 studies, reported that the overall proportion of HCW who were SARS-CoV-2 positive among all COVID-19 patients was 10.1% [5]. In Spain, 20.4% of confirmed COVID-19 cases were HCW [6].

In Scotland, HCW and their households contributed a sixth of COVID-19 admissions to hospital [7]. In Spain and USA, 10% and 8% of HCW with COVID-19 were hospitalized respectively [6, 8]. Young women nurses were more frequently infected [9–11]. Comorbidities were frequent in HCW hospitalized with COVID-19, particularly diabetes, hypertension, obesity, asthma and immunodepression [6, 10, 11].

There is still controversy over the risk of death in HCW with COVID-19. While it is high in Mexico, it is low in Germany and Malaysia [12, 13]. The main objectives of this study were to describe the clinical characteristics and outcomes of HCW hospitalised in Spain due to SARS-CoV-2 infection, and to determine if working in healthcare is associated with higher rates of complications and mortality.

## Methods

### Study design and population

The Sociedad Española de Medicina Interna–COVID-19 Registry (SEMI-COVID-19; SEMI is the acronym in English of Spanish Society of Internal Medicine) is an ongoing, nationwide, multicentre, observational retrospective registry, participated by 150 hospital centres throughout Spain. Detailed features of the registry have been reported elsewhere [14–16]. A total of 10,600 consecutive patients were recruited from March 1, 2020 to May 22, 2020.

### Inclusion criteria

The SEMI-COVID-19 Registry includes patients > 18 years admitted to hospital with COVID-19 confirmed microbiologically by reverse transcription polymerase chain reaction (RT-PCR) testing of a nasopharyngeal swab sample, sputum specimen or bronchoalveolar lavage. The exclusion criteria were subsequent admission of the same patient or denial or withdrawal of informed consent. This study analyses the subpopulation of patients between 20 and 65 years of age. In Spain, 20 years is the youngest possible age for working in healthcare and 65 years is the retirement age. HCW were defined as physicians, nurses, nurse aides, and non-healthcare professionals such as the administrative and cleaning staff of hospitals and healthcare centres. In the workplace, all HCW were wearing personal protective equipment and had been educated about infection protection.

### Procedures and variables

Admission and treatment of patients took place at the discretion of the attending physicians based on their clinical judgment, local protocols, and the updated recommendations of the Spanish Ministry of Health. The technical report of the Spanish Agency of Medicines and Medical Devices recommended lopinavir/ritonavir 400/100 mg bid, hydroxychloroquine 400 mg bid the first day and 200 mg bid after [17]. Other drugs as remdesivir, tocilizumab or interferon beta-1B were used in clinical trials or as compassionate use.

Data were collected retrospectively in an online electronic data capture system and were extracted from electronic health records. The access to database to obtain the data used in this study was on May 22th, 2020. Approximately 300 variables were collected including epidemiological data, RT-PCR data, personal medical and medication history, symptoms and physical examinations findings at admission, laboratory and diagnostic imaging tests, pharmacological treatment and ventilator support during hospitalization, complications and death during hospitalization, and readmissions and survival 30 days after diagnosis. Comorbidity was assessed using the Charlson Comorbidity Index (CCI) [18]. This index includes 19 diseases and each of them is assigned a weight from 1 to 6. A score of 0–1 is considered low comorbidity, 2 moderate comorbidity and ≥ 3 high comorbidity. The Barthel index was used to measure ability to carry out basic daily living tasks in 10 areas: feeding, bathing, dressing, grooming, bladder control, bowel control, toilet use, transferring, moving on level surfaces, and walking up and downstairs [19]. Its scores range between 0 and 100: the higher the score the more independent the person. A patients is considered independent or mild dependent if his or her score is ≥ 90, moderate dependent if is 60–90, and severe dependent if is <60. Obesity was defined as body mass index >30 kg/m^2^.

The main endpoint was mortality during admission. Intensive care unit (ICU) admission, days in the ICU, invasive or non-invasive ventilation, all-cause re-admission, 30-days all-cause mortality, and length-of-stay were secondary endpoints.

### Ethical aspects

The study was carried out in accordance with the Declaration of Helsinki. The processing of personal data strictly complied with Spanish Law 14/2007, of July 3, on Biomedical Research and Spanish Organic Law 3/2018, of 5 December, on the Protection of Personal Data and the Guarantee of Digital Rights. The study was approved by the Provincial Research Ethics Committee of Málaga (Spain) following the recommendation of the Spanish Agency of Medicines and Medical Products (AEMPS, for its initials in Spanish). All patients—or their caregivers, in the event they presented with cognitive impairment—gave their informed oral consent. The consent was witnessed by the patient´s relatives and a nurse.

### Statistical analysis

The patients were divided into two groups: HCW and non HCW (NHCW). Continuous variables were tested for normal distribution with the Kolmogorov-Smirnov test. Quantitative variables are expressed as mean (standard deviation, SD) or median [interquartile range]. Comparisons between groups were made using Student’s t-test and the Mann-Whitney U test. Categorical variables are expressed as absolute frequencies and percentages. Comparisons between them were made using the chi-square test with the Yates correction and with the Fisher’s exact test when necessary.

Two multivariate logistic regression models were performed to analyse the association between working in healthcare and occurrence of sepsis and mortality. The first model included age, sex, ethnicity, CCI score, and healthcare working. The second model included the previous variables and added the comorbidities with a statistical significance p<0.1 in the univariate model.

In all cases, statistical significance was established as p<0.05. Statistical analysis was carried out using Statistical Package for the Social Sciences (SPSS) 21.0 software for Windows.

## Results

**Fig 1:**
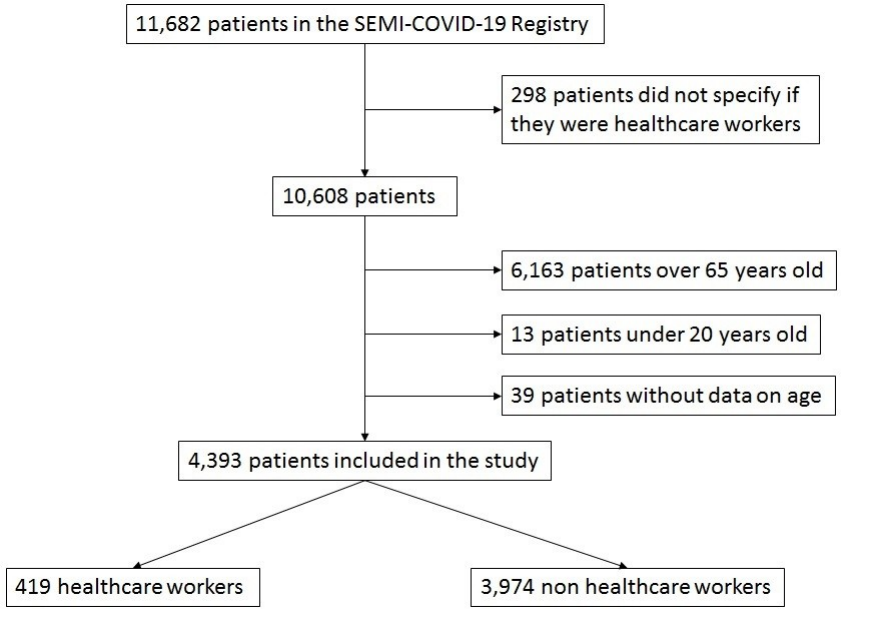
Patient inclusion flowchart.

**Fig 1** shows the flowchart for patient inclusion. A total of 4,393 patients were included, of which 419 (9.5%) were HCW. Among HCW, 142 (33.9%) were medical doctors, 107 (25.5%) were nurses, 98 (23.4%) were nurse aides, and 72 (17.2%) held other positions within healthcare. The departments that most infected patients worked in were primary care (16.6%), the emergency department (11.3%), and internal medicine (11.3%).

### Demographic and clinical characteristics

Baseline demographic and clinical characteristics and comorbidities are shown in **Table 1**. HCW were more often Caucasian women, and reported more frequent contact with a COVID-19 patient (57.8% vs. 22.1%, p<0.001). HCW reported more frequently nosocomial infection (41.4% vs. 3.6%, p<0.001). Moderate and severe dependence was more frequent in NHCW. There was no difference in comorbidity measured as Charlson index score, but the prevalence of comorbidities as alcohol use disorder, hypertension, dyslipidemia, obesity, diabetes, myocardial infarction, stroke, dementia, chronic obstructive pulmonary disease, obstructive sleep apnea-hypopnea syndrome, chronic kidney disease and malignancy was higher in NHCW.

**Table 1:** Sociodemographic characteristics, comorbidities and treatment of patients included.

	Total	NHCW	HCW	p
	(n = 4393)	(n = 3974)	(n = 419)	
Age, years *(n = 4393)*	53 (15)	53 (15)	52 (15)	0.439
Gender, female *(n = 4386)*	1791 (40.8)	1530 (38.6)	261 (62.4)	<0.001
Race/ethnicity *(n = 4303)*				
Caucasian	3399 (79.0)	3038 (78.1)	361 (87.2)	<0.001
BAME people	904 (21.0)	851 (21.91)	53 (12.8)	
**Infection route** *(n = 4374)*				
Out-of-hospital	3978 (90.9)	3759 (94.9)	219 (53.0)	
Nosocomial	315 (7.2)	144 (3.6)	171 (41.4)	<0.001
Nursing home	81 (1.9)	58 (1.5)	23 (5.6)	
**Dependency** *(n = 4351)*				
Independent or mild	4195 (96.4)	3779 (96.1)	416 (99.5)	
Moderate	77 (1.8)	75 (1.9)	2 (0.5)	0.001
Severe	79 (1.8)	79 (2.0)	0 (0.0)	
**Tobacco use** *(n = 4191)*				
Current smoker	289 (6.9)	266 (7.0)	23 (5.6)	0.291
Alcohol use disorder *(n = 4278)*	190 (4.4)	187 (4.8)	3 (0.7)	<0.001
Comorbidity Charlson Comorbidity Index *(n = 4274)*	0 (1)	0 (1)	0 (1)	0.447
Low	3643 (85.2)	3268 (84.5)	375 (91.9)	
Moderate	33 (7.7)	3.9 (8.0)	21 (5.1)	<0.001
High	301 (7.0)	289 (7.5)	12 (2.9)	
**Comorbidities**				
Hypertension *(n = 4388)*	1116 (25.4)	1044 (26.3)	72 (17.2)	<0.001
Dyslipidaemia *(n = 4389)*	1019 (23.2)	946 (23.8)	73 (17.1)	0.003
Obesity *(n = 4005)*	872 (21.8)	803 (22.2)	698 (17.6)	0.037
Diabetes mellitus *(n = 4374)*	457 (10.4)	434 (11.0)	23 (5.5)	0.001
AMI *(n = 4387)*	76 (1.7)	75 (1.9)	1 (0.2)	0.014
Heart failure *(n = 4387)*	64 (1.5)	61 (1.5)	64 (1.5)	0.182
Atrial fibrillation *(n = 4382)*	86 (2.0)	83 (2.1)	3 (0.7)	0.054
Stroke/TIA *(n = 4378)*	96 (2.2)	94 (2.4)	2 (0.5)	0.012
Dementia *(n = 4381)*	47 (1.1)	47 (1.2)	0 (0)	0.025
COPD *(n = 4384)*	99 (2.3)	99 (2.5)	0 (0)	0.001
OSAHS *(n = 4362)*	232 (5.3)	223 (5.7)	9 (2.2)	0.002
Moderate-severe CKD *(n = 4382)*	97 (2.2)	94 (2.4)	3 (0.7)	0.028
Moderate-severe CLD *(n = 4387)*	36 (0.8)	36 (0.9)	0 (0)	0.050
Malignancy *(n = 4363)*	254 (5.8)	239 (6.1)	15 (3.6)	0.042
**Biochemistry**				
Glucose, mg/dL *(n = 4231)*	105 (29)	105 (10)	102 (24)	0.027
Creatinine mg/dL *(n = 4355)*	0.83 (0.31)	0.84 (0.15)	0.76 (0.35)	<0.001
Urea, mg/dL *(n = 3336)*	29 (15)	29 (6)	26 (11)	<0.001
LDH, U/L *(n = 3887)*	304 (171)	299 (63)	273 (127)	<0.001
AST, U/L *(n = 3470)*	36 (27)	37 (11)	30 (21)	<0.001
ALT, U/L *(n = 4191)*	35 (30)	35 (12)	30 (25)	<0.001
CRP, mg/L *(n = 4355)*	41.8 (89)	49 (23)	27.8 (64.7)	<0.001
Serum ferritin, mcg/L *(n = 1629)*	643 (1028)	669 (364)	388 (779)	0.001
D-dimer, ng/mL *(n = 3485)*	550 (544)	490 (199)	410 (431)	0.001
**Blood count**				
Haemoglobin, g/dL *(n = 4368)*	14.3 (2)	14.4 (2)	14.2 (1.6)	0.044
WBC, x10^6^/L *(n = 4370)*	5900 (3400)	5910 (1310)	5530 (2840)	0.005
Lymphocytes *(n = 4365)*	1000 (610)	1000 (245)	1072 (600)	0.261
Platelets *(n = 4373)*	200500 (103250)	196000 (42000)	187000 (90000)	0.103
**Radiological findings**				
Bilateral condensation *(n = 4352)*	1,378 (31.7)	1267 (32.2)	111 (26.7)	0.033
Bilateral interstitial infiltrates *(n = 4355)*	2322 (53.3)	2128 (54.0)	194 (46.7)	0.018
Pleural effusion *(n = 4356)*	104 (2.4)	101 (2.5)	3 (0.7)	0.047
**Treatments**				
LPV/r *(n = 4357)*	3096 (71.1)	2,803 (71.0)	291 (71.3)	0.913
Interferon Beta-1B *(n = 4329)*	567 (13.1)	520 (13.3)	47 (11.5)	0.312
Remdesivir *(n = 4315)*	27 (0.6)	21 (0.5)	6 (1.5)	0.023
Hydroxychloroquine *(n = 4366)*	3916 (89.7)	3531 (89.3)	385 (93.0)	0.020
Tocilizumab *(n = 4349)*	462 (10.6)	413 (10.5)	49 (12.0)	0.359
Systemic corticosteroids *(n = 4346)*	1228 (28.3)	1121 (28.5)	107 (26.0)	0.279

ALT: alanine aminotransferase; AMI: acute myocardial infarction; AST: aspartate aminotransferase; BAME: black, Asian and minority ethnic; CLD: chronic liver disease; CCI: Charlson Comorbidity Index; CKD: chronic kidney disease; COPD: chronic obstructive pulmonary disease; CRP: C-reactive protein; LDH: lactate dehydrogenase; OSAHS: obstructive sleep apnoea/hypopnoea syndrome; TIA: transient ischemic attack, WBC: white blood cell.

Data are expressed as median (interquartile range) and n (%).

The median time from first symptoms to admission was 7 [5–9] days, without difference between HCW and NHCW. There were some differences in symptoms and physical examination findings. Dry cough (72.3% vs. 67.3%, p = 0.003), asthenia (54.6% vs. 44.8%, p<0.001), arthralgia (48.1% vs. 39.2%, p<0.001), ageusia (14.6% vs. 9.4%, p = 0.001) and anosmia (14.6% s. 8.7%, p<0.001) were more frequent in HCW, and temperature ≥ 38°C (74.4% vs. 68,5%, p = 0.007) and oxygen saturation ≤ 92% (24.2% vs 11.8%, p<0.001) in NHCW.

At admission the levels of serum glucose, creatinine, urea, lactate dehydrogenase, aspartate aminotransferase, alanine aminotransferase, C-reactive protein, ferritin, D-dimer, hemoglobin and the count of white blood cells were lower in HCW. NHCW presented more frequently severe radiological findings—i.e. pleural effusion, bilateral condensation, and bilateral interstitial infiltrates (all p≤0.025).

### Treatments

There were no differences in the treatment for COVID-19 disease between HCW and NHCW except for hydroxychloroquine and remdesivir (93% vs. 89.3%; p = 0.02 and 1.5% vs. 0.5%; p = 0.0.23 respectively).

### Outcomes

**Table 2** shows the outcomes. Sepsis was more frequent in NHCW (3.9% vs. 1.7%; p = 0.024). Age, obesity, stroke and moderate-severe chronic kidney disease were associated with the occurrence of sepsis (**Table 3**). There were no differences in the occurrence of other complications, the need of respiratory support or ICU admission. The length of hospital stay was 8 (7) days without difference among HCW and NHCW.

**Table 2:** Outcomes.

	Total	NHCW	HCW	p
**Complications**				
Bacterial pneumonia *(n = 4356)*	330 (7.6)	305 (7.7)	25 (6.0)	0.209
ARDS *(n = 4355)*	1001 (23.0)	919 (23.3)	82 (19.8)	0.101
Acute kidney failure *(n = 4353)*	243 (5.6)	228 (5.8)	15 (3.6)	0.068
Sepsis *(n = 4351)*	160 (3.7)	153 (3.9)	7 (1.7)	0.024
Shock *(n = 4349)*	132 (3.0)	125 (3.2)	7 (1.7)	0.094
Thromboembolic disease *(n = 4350)*	71 (1.6)	64 (1.6)	7 (1.7)	0.921
**Respiratory support**				
High flow nasal cannula *(n = 4334)*	327 (7.5)	293 (7.5)	34 (8.2)	0.578
Noninvasive mechanical ventilation *(n = 4357)*	174 (4.0)	156 (4.0)	18 (4.3)	0.707
Invasive mechanical ventilation *(n = 4357)*	314 (7.2)	291 (7.4)	23 (5.5)	0.168
**Intensive care unit (ICU)**				
Admission to ICU *(n = 4385)*	415 (9.5)	371 (9.4)	44 (10.5)	0.435
Days in the ICU	11 (10)	11 (11)	8.5 (10)	0.099
**Death and readmission**				
Hospital length-of-stay, days *(n = 4392)*	8 (7)	8 (8)	7 (7)	0.067
In-hospital death *(n = 4393*	194 (4.4)	191 (4.8)	3 (0.7)	<0.001
Readmission *(n = 4194)*	121 (2.9)	112 (2.9)	9 (2.3)	0.449

ARDS: acute respiratory distress syndrome; ICU: intensive care unit

Data are expressed as n (%) and median [interquartile range]

**Table 3:** Factors associated with the occurrence of sepsis.

	Univariate		Multivariate	
Variable	OR (95%CI)	p	OR (95%CI)	p
**Model 1**				
Age	1.047 (1.027–1.067)	<0.001	1.041 (1.021–1.062)	<0.001
Male sex	1.687 (1.194–2.383)	0.003	1.627 (1.140–2.324)	0.007
BAME	0.981 (0.664–1.450)	0.923		
HCW	0.427 (0.199–0.916)	0.029	0.458 (0.200–1.049)	0.065
Comorbidity				
Low	reference		reference	
Moderate	2.399 (1.508–3.817)	<0.001	2.043 (1.276–3.273)	0.003
High	2.258 (1.382–3.692)	0.001	1.782 (1.080–2.942)	0.024
**Model 2**				
Age	1.047 (1.027–1.067)	<0.001	1.040 (1.018–1.062)	<0.001
Male sex	1.687 (1.194–2.383)	0.003	1.347 (0.919–1.973)	0.126
BAME	0.981 (0.664–1.450)	0.923		
HCW	0.427 (0.199–0.916)	0.029	0.506 (0.219–1.167)	0.110
Alcohol	2.232 (1.262–3.945)	0.006	1.624 (0.848–3.113)	0.144
Smoking	2.024 (1.232–3.327)	0.005	1.688 (0.961–2.966)	0.069
Hypertension	1.476 (1.054–2.066)	0.023	0.936 (0.617–1.421)	0.756
Dyslipidemia	1.486 (1.054–2.096)	0.024	0.754 (0.485–1.173)	0.211
Obesity	1.645 (1.148–2.357)	0.007	1.552 (1.035–2.327)	<0.001
Diabetes	1.795 (1.172–2.747)	0.007	0.929 (0.533–1.617)	0.794
AIM	1.896 (0.755–4.765)	0.173		
Heart failure	1.763 (0.633–4.912)	<0.001		
Atrial fibrillation	1.632 (0.652–4.085)	0.295		
Stroke/TIA	3.209 (1.633–6.303)	0.001	2.272 (1.044–4.945)	0.039
Dementia	3.932 (1.645–9.402)	0.002	1.703 (0.646–5.811)	0.238
COPD	3.123 (1.591–6.129)	0.001	1.405 (0.489–5.933)	0.403
OSAHS	2.241 (1.330–3.776)	0.002	1.701 (0.937–3.089)	0.081
Moderate-severe CKD	2.837 (1.401–5.746)	0.004	2.669 (1.235–5.767)	0.013
Moderate-severe CLD	3.328 (1.163–9.525)	0.025	1.639 (0.506–5.302)	0.410
Malignancy	0.730 (0.338–1.574)	0.422		

AIM: acute myocardial infarction BAME: black, Asian and minority ethnic; CCI: Charlson Comorbidity Index score; CI: confidence interval; CKD: chronic kidney disease; CLD; chronic liver disease; COPD: chronic obstructive pulmonary disease; HCW: healthcare workers; OR: odds ratio; OSAHS: obstructive sleep apnoea/hypopnoea syndrome; TIA: transient ischemic attack

**Fig 2:**
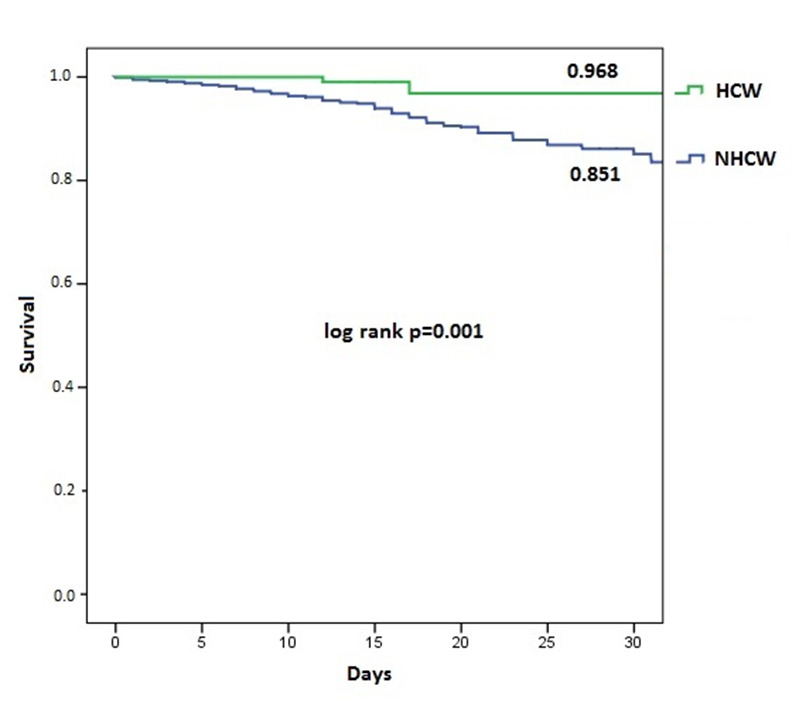
30-days Kaplan-Meier survival curves.

During hospitalization 194 (4.4%) patients died. In-hospital mortality was lower in HCW (0.7% vs 4.8%; p<0.001). The readmission rate was 2.9%. Half of readmissions were due to COVID-19 disease (**Table 2**). The 30-days survival was 96.8% in HCW and 85.1% in NHCW (p = 0.001). **Fig 2** shows the Kaplan-Meier survival curve.

The factors associated with in-hospital mortality are shown in **Table 4**. In the first multivariate analysis model, age, male sex and Charlson Comorbidity index score were associated with higher in-hospital mortality and healthcare working with lower mortality (OR 0.219, 95%CI 0.069–0.693, p = 0.01). In the second model, including comorbidities, healthcare working was also associated with a lower in-hospital mortality (OR 0.285 95%CI 0.089–0.908; p = 0.034).

**Table 4:** Factors associated with mortality.

	Univariate		Multivariate	
Variable	OR (95%CI)	p	OR (95%CI)	p
**Model 1**				
Age	1.073 (1.053–1.095)	<0.001	1.061 (1.039–1.083)	<0.001
Male sex	1.884 (1.366–2.599)	<0.001	1.927 (1.369–2.711)	<0.001
BAME	0.743 (0.505–1.094)	0.132		
HCW	0.143 (0.045–0.449)	0.001	0.211 (0.067–0.667)	0.008
Comorbidity				
Low	reference		reference	
Moderate	2.799 (1.796–4364)	<0.001	2.246 (1.429–3.531)	<0.001
High	6.995 (4.914–9.956)	<0.001	5.205 (3.614–7.498)	<0.001
**Model 2**				
Age	1.073 (1.053–1.095)	<0.001	1.055 (1.031–1.081)	<0.001
Male sex	1.884 (1.366–2.599)	<0.001	1.712 (1.159–2.530)	0.007
BAME	0.743 (0.505–1.094)	0.132		
HCW	0.143 (0.045–0.449)	0.001	0.285 (0.089–0.908)	0.034
Alcohol	2.642 (1.604–4.351)	<0.001	1.266 (0.674–2.379)	0.463
Smoking	2.491 (1.625–3.820)	<0.001	2.436 (1.473–4.030)	0.001
Hypertension	2.362 (1.762–3.166)	<0.001	1.360 (0.923–2.004)	0.120
Dyslipidemia	1.886 (1.393–2.552)	<0.001	0.819 (0.543–1.236)	0.342
Obesity	2.029 (1.471–2.798)	<0.001	1.679 (1.136–2.481)	0.009
Diabetes	2.870 (2.030–4.059)	<0.001	1.464 (0.926–2.316)	0.103
AIM	2.609 /1.236–5.508)	0.012	1.278 (0.518–3.156)	0.595
Heart failure	3.660 (1.782–7.519)	<0.001	1.158 (0.426–3.147)	0.773
Atrial fibrillation	2.279 (1.085–4.788)	0.03	0.939 (0.376–2.344)	0.893
Stroke/TIA	3.913 (2.178–7.033)	<0.001	0.952 (0.417–2.171)	0.907
Dementia	8.773 (4.551–16.910)	<0.001	8.884 (3.800–20.772)	<0.001
COPD	6.017 (3.600–10.057)	<0.001	2.330 (1.233–4.403)	0.009
OSAHS	3.165 (2.058–4.867)	<0.001	1.659 (0.955–2.884)	0.072
Moderate-severe CKD	5.351 (3.138–9.126)	<0.001	3.649 (1.889–7.051)	<0.001
Moderate-severe CLD	5.375 (2.324–12.429)	<0.001	1.845 (0.657–5.180)	0.245
Malignancy	3.710 (2.490–5.527)	<0.001	3.058 (1.891–4.943)	<0.001

AIM: acute myocardial infarction; BAME: black, Asian and minority ethnic; CCI: Charlson Comorbidity Index score; CI: confidence interval; CKD: chronic kidney disease; CLD; chronic liver disease; COPD: chronic obstructive pulmonary disease; HCW: healthcare workers; OR: odds ratio; OSAHS: obstructive sleep apnoea/hypopnoea syndrome; TIA: transient ischemic attack

## Discussion

The main findings of our study were that hospitalised HCW had less severe COVID-19 and lower mortality.

The demographic characteristics of our patients were consistent with other reports [14, 20, 21]. Worldwide, men were more likely to be infected by SARS-CoV-2 than women. However, among HCW, women were the most affected [8, 9, 13, 22]. We think that this difference is due to the higher proportion of females in healthcare professions. When we compared our HCW cohort with those reported in other studies, our patients were more than ten years older [8–10, 13, 22, 23]. This could be due to the different inclusion criteria used in each study. In the USA, Hughes et al. [8] reported on the characteristics of 100,570 HCW with a median age of 41 years, of whom only 6832 were hospitalised. Wang et al included 80 HCW hospitalised in Wuhan, of whom 57 were confirmed cases and 23 were clinical diagnosis [11]. In our cohort, only hospitalised patients between 20 and 65 years old with a confirmed diagnosis of COVID-19 were included, while the rest included all HCW with COVID-19.

An interesting finding in our study was that upon admission HCW presented milder symptoms, such as loss of smell or taste and arthralgia, less severe radiological findings and lower lactate dehydrogenase, C-reactive protein, serum ferritin and D-dimer levels. And all of it even though there was no difference between HCW and NHCW in time from onset of symptoms and admission. There was not an explanation for this, but we hypothesize that it could be due to HCW were hospitalised earlier and more easily than NHCW.

ARDS is overwhelmingly the main cause of death in hospitalised COVID-19 patients. In our study, sepsis was less frequent in HCW but there was no difference in ARDS, the rest of complications, the need of respiratory support nor the ICU admission. In-hospital and 30-days mortality were lower in HCW. In a systematic review Similar results were reported in the systematic review by Sahu et al. [5]. A healthy worker effect could explain these results. Severily ill and chronically disabled are ordinarily excluded from employment [24]. The difference observed in prevalence of comorbidities between HCW and NHCW supports this explanation. The better clinical and analytical profile of the HCW at admission may be due to their knowledge of mild symptoms of COVID-19 and their ability to identify them in themselves. In this regard, increased education on the earliest and mildest symptoms of COVID-19 could help NHCW to recognize and to report them to a healthcare centre earlier in the course of their disease.

There are geographical differences in mortality observed in hospitalised COVID-19 HCW. In a teaching hospital in Belgium the mortality of HCW was 0.5% [22]. In a single-centre study in Wuhan, in-hospital death in HCW with confirmed SARS-CoV-2 infection was 1.7%, more than twice in our study [11]. The mortality in a multicentre study in New York City was 21%, far higher than that we observed [21]. Also, high mortality, 14.7%, was reported in Brazil [10]. In Mexico, a mortality of 2% of HCW with COVID-19 was reported [23]. Their mortality was higher than ours even though only 9% of them needed to be hospitalized. The authors explain their high mortality because of different reasons. On the one hand, in Mexico there is a high prevalence of comorbidities which are associated with severe COVID-19. On the other hand, due to structural inequalities, their healthcare system is highly heterogeneous and there is a remarkable amount of marginalized communities. Therefore, the prevalence of comorbidities, the level of economic wealth, and the organization of healthcare in the different countries could explain these differences observed in the mortality.

Several studies have reported that age, male sex and comorbidity were associated with higher mortality in COVID-19 patients [25–28]. However the research about healthcare working as risk factor of mortality is scarce. HCW worry and are afraid to be infected and die for COVID-19. Our results confirm that COVID-19 is less severe and leads to less mortality in HCW. This is one of the novel contributions of our study that has evident clinical, epidemiological and occupational health implications.

Among the strengths of our study are its multicentre design, the inclusion of patients from the entire country, and the large number of patients included, which provides an adequate statistical power to confirm hypotheses. However, our study also has limitations. Only hospitalised patients were included, so it is not possible to extrapolate our results to non-hospitalised patients. The large number of researchers involved and variability in the availability of data in each hospital could have led to information bias. Finally, the voluntary participation of each centre could have caused selection bias.

## Conclusions

HCW had fewer comorbidities, milder symptoms, and a better prognosis than the NHCW. Our results suggest that professional exposure to COVID-19 in HCW does not lead to greater clinical severity nor mortality.
